# Colchicine treatment in PFAPA: how long should we wait for the clinical response?

**DOI:** 10.1007/s00431-025-06158-w

**Published:** 2025-05-02

**Authors:** Lutfiye Koru, Elif Selcen Yabanci Erten, Hatice Kubra Dursun, Eda Nur Dizman, Feray Kaya, Elif Kucuk, Zelal Aydin, Merve Ozen Balci, Kubra Ozturk, Fatih Haslak

**Affiliations:** 1 Department of Pediatric Rheumatology, Istanbul Medeniyet University Istanbul, Istanbul, Turkey; 2 Department of Pediatrics, Istanbul Medeniyet University, Istanbul, Turkey

**Keywords:** Fever, Stomatitis, Aphthous, Colchicine, Therapeutics

## Abstract

We aimed to evaluate the efficacy of prophylactic colchicine treatment in periodic fever, aphthous stomatitis, pharyngitis, and cervical adenitis (PFAPA) using the Auto-Inflammatory Diseases Activity Index (AIDAI) score measured before and during treatment. We also aimed to determine the optimal treatment duration for observing clinical response and to identify factors predicting response to treatment. Children with PFAPA receiving colchicine were included in the study. We utilized AIDAI scoring system by evaluating the sore throat, tonsillitis, and oral aphthae under the general symptoms heading. This form of AIDAI was given to the families, and they were asked to document their symptoms on a daily basis. AIDAI scores were calculated for the month before colchicine initiation and for the first and third months after. Data were obtained retrospectively. The mean age of patients was 4.4 ± 2.13 years, and almost two third of them (50/76) were male. The difference in AIDAI scores between the month before colchicine treatment and the 1st month after the treatment was significant, while the difference between the 1st and 3rd month after the treatment was not (*p* = 0.002 vs. *p* = 0.463). Patient treatment responses were: 44 (57.9%) complete response, 15 (19.7%) partial response, and 17 (22.4%) unresponsive to colchicine. *Conclusion*: Based on this finding, we can conclude that in the case of partial response to colchicine treatment after the first month, dose escalation, review of treatment compliance and the need for prophylaxis should be reevaluated.

**Table:** 

**What is known:** • *Colchicine prophylaxis is recommended in PFAPA patients; however, the optimal time point for response assessment remains unclear*. • *The AIDAI score is suggested for measuring disease activity in autoinflammatory conditions*.	
**What is new:** • *This is the first repeated-measure study evaluating colchicine response in PFAPA patients using the AIDAI score*. • *Clinical response predominantly emerged within the first month, suggesting this period may be critical for evaluation*.

**Supplementary Information:**

The online version contains supplementary material available at 10.1007/s00431-025-06158-w.

## Introduction

Periodic fever, aphthous stomatitis, pharyngitis, and cervical adenitis (PFAPA) is the one of the most prevalent form of autoinflammatory diseases (AIDs) in pediatric populations. This condition typically manifests in early childhood with recurrent episodes of fever that persist for 3 to 7 days and occur at intervals of 2 to 8 weeks. During these episodes, pharyngitis, cervical lymphadenopathy, and/or aphthous stomatitis may be observed. Between febrile episodes, children exhibit no symptoms or signs and have normal growth and development. PFAPA usually resolves on its own after several years, but the impact on the quality of life for both affected children and their families cannot be ignored [1–4].

The lack of sufficient clinical studies has resulted in no established treatment guidelines for PFAPA, and therapeutic approaches are predominantly based on expert opinion, resulting in significant variation among clinical practices [5, 6]. However, current treatment recommendations include corticosteroids given at the onset of the attack, daily prophylaxis particularly with colchicine, and tonsillectomy [7, 8].

Despite the existence of several studies evaluating colchicine response in PFAPA, the criteria employed to assess treatment response vary across these studies [9–12]. Although it is the only suggested tool for measuring disease activity in PFAPA patients, only one study has evaluated Auto-Inflammatory Diseases Activity Index (AIDAI) scores for assessing the colchicine responses of these patients [13, 14].

We aimed to evaluate the efficacy of prophylactic colchicine treatment in PFAPA patients using the AIDAI score measured before and during treatment. We also aimed to determine the optimal treatment duration for observing clinical response and to identify factors predicting response to treatment.

## Materials and Methods

### Patients and data collection

The study examined 130 children admitted to our tertiary care center between 2022 and 2024, who were classified as PFAPA by a pediatric rheumatologist according to EUROFEVER/PRINTO criteria [15]. Families were offered the treatment options recommended by international consensus [8]. The study included 76 patients who adhered to colchicine prophylaxis at the dosage recommended by international consensus and received colchicine for a minimum duration of three months [8]. Demographic data, clinical and laboratory findings in the attack before and after colchicine prophylaxis, duration and doses of colchicine were obtained retrospectively from patient records.

This study was conducted in compliance with the Helsinki Declaration as well as local laws and regulations. Informed consent was obtained from the patients and their legal caregivers. The ethics committee of a tertiary center approved our study (18/10/2024/725).

### Evaluation of treatment response and influencing factors

AIDAI scores were suggested to be a useful tool for assessing disease activity in PFAPA patients [14]. We utilized AIDAI scoring system by evaluating the sore throat, tonsillitis, and oral aphthae under the general symptoms heading. This form of AIDAI was given to the families, and they were asked to document their symptoms on a daily basis (Supplementary Table 1). AIDAI scores were calculated for the month preceding the initiation of colchicine treatment and for the first- and third-months following colchicine treatment. Patients were compared in terms of demographic and clinical characteristics as well as Mediterranean Fever *(MEFV*) gene analysis results in order to determine the factors affecting the difference between AIDAI scores before and after prophylaxis. The clinical manifestations of the attack were analysed both individually and categorised as gastrointestinal tract manifestations (nausea and vomiting, abdominal pain, diarrhoea, constipation) and constitutional features (myalgia, fatigue, arthralgia). Utilizing the data derived from these comparative analyses, predictive factors for the treatment response were identified. Following a three-month observation period subsequent to the initiation of prophylaxis, individuals who exhibited no symptoms during the final month were classified as having achieved a complete response to colchicine. Those who demonstrated a reduction of ≥ 50% in clinical findings were considered to have a partial response, whereas individuals with less reduction, stabilization, or worsening of symptoms were categorized as unresponsive.

### Statistical analysis

We performed the statistical analysis using SPSS for Windows, version 26.0 (SPSS Inc., Chicago, IL). Kolmogorov- Smirnov test was used to assess the distribution of continuous variables. While those with a normal distribution were presented as mean ± standard deviation, those distributed abnormally were presented as median (minimum–maximum). The chi-square test or Fisher’s exact test was used to compare the categorical variables, expressed as numbers (percentages). Mann–Whitney U test or Student’s t-test was used to compare the continuous variables when appropriate.

To evaluate the change in AIDAI scores before and after prophylaxis, the logarithm of the abnormally distributed AIDAI scores was initially calculated to transform the data to fit a normal distribution. A repeated measures ANOVA test was then performed. Furthermore, the possible effects of baseline clinical and laboratory variables on the AIDAI changing pattern during our observation period were assessed by multivariate ANOVA test.

The study focused on the period during which the decline in AIDAI scores was most pronounced, specifically from one month prior to prophylaxis to the first month following prophylaxis (Delta AIDAI, a normally distributed continuous variable). A Student’s t-test was employed to compare the Delta AIDAI across categorical variables. To evaluate the correlation between the Delta AIDAI and other continuous variables, Pearson or Spearman correlation tests were utilized as appropriate. The impact of demographic, clinical, and laboratory data on the Delta AIDAI was analyzed using linear regression analysis. Variables identified as significant in the Student’s t-tests and correlation tests, along with confounding factors (The presence of gastrointestinal symptoms during the attack, age, and gender), were incorporated into the model.

Kaplan–Meier analysis was conducted to evaluate the time interval between the first attack and the initiation of colchicine treatment, and to compare these intervals between individuals with and without the M694 V variation on the *MEFV* gene.

The Wilcoxon test was employed to compare the maximum temperature and duration of the last attacks preceding the initiation of treatment with those recorded during the first attacks within the three-month period following the commencement of treatment, which constituted the observation period. Statistical significance was defined as *p* < 0.05. Prism software (Prism 8, GraphPad Software, San Diego, California) was utilized to generate graphical representations of the data.

## Results

### Patient characteristics and genetic findings

The mean age of 76 patients with PFAPA syndrome was 4.4 ± 2.13 years and 65.8% (n = 50) was male. The median age at the time of the first attack and when colchicine prophylaxis was initiated was 2.5 (0.25–7.5) and 4.29 (1.08–9.41) years, respectively. Familial history of recurrent fever was observed in 63.2% (n = 48) of the patients. Additionally, 30 patients (39.5%) reported a family history of tonsillectomy. Before prophylaxis, the median duration of attacks was 4 (2–10) days and the median interval between attacks was 22.50 (10–90) days. The most common symptom during the attack was fever which was observed in all patients, followed by sore throat (n = 75, 98.7%), lymphadenitis (n = 56, 73.7%), and oral aphthae (n = 42, 55.3%). The median C-reactive protein (CRP), and the mean erythrocyte sedimentation rate (ESR) levels were 46.9 (7.9–222.6) mg/L and 35 ± 16.2 mm/hour during the attack, respectively. *MEFV* gene analysis was available in 84.2% (n = 64) of the patients. Heterozygous variation in the *MEFV* gene was detected in 42.1% (n = 32) of the patients. The most common heterozygous variation in the *MEFV* gene was found in exon 10 (n = 17, 27%). The most common *MEFV* variant was M694 V (n = 11, 17.2%).

### Comparison of characteristics of patients with and without MEFV variation

When patients with and without *MEFV* variation were compared, no statistically significant difference was found in terms of demographic and clinical characteristics. The results are summarized in Table 1.

**Table 1:** Comparison of clinical and demographic characteristics of patients with and without MEFV variation

	Patients with *MEFV* variation (n = 32)	Patients without *MEFV* variation (n = 32)	*p* value
Male gender (*n*,%)	20 (31.2%)	20 (31.2%)	1.00
Age at diagnosis (Year) (Mean ± SD)	4.87 ± 2.27	4.14 ± 2.09	0.189
Colchicine starting age (Year) (Median (Min–Max))	4.41 (1.16–6.6)	4.25 (1.08–9.41)	0.416
Number of monthly attacks before prophylaxis (Median (Min–Max))	1 (0–5)	1 (0–3)	0.721
Duration of attack before prophylaxis (Day) (Median (Min–Max)	4 (2–10)	5 (3–10)	0.252
Highest fever value in the attack before prophylaxis (^0^C) (Median (Min–Max)	40 (39–41)	40 (38.6–41)	0.920
Time between attacks before prophylaxis (Day) (Median (Min–Max)	30 (10–60)	20 (10–90)	0.594
Clinical findings in an attack			
* Sore throat in an attack before prophylaxis (n,%)*	31 (96.8%)	32 (100%)	1.00
* Oral aphthae in pre-prophylaxis attack (n,%)*	17 (53.1%)	21 (65.6%)	0.309
* Lymphadenitis in an attack before prophylaxis (n,%)*	22 (68.7%)	26 (81.2%)	0.248
* Abdominal pain in the attack before prophylaxis (n,%)*	21 (65.6%)	19 (59.3%)	0.606
* Headache in an attack before prophylaxis (n,%)*	9 (28.1%)	8 (25%)	0.777
* Chest pain in an attack before prophylaxis (n,%)*	1 (3.1%)	2 (6.2%)	0.554
* Arthralgia in the attack before prophylaxis (n,%)*	14 (43.7%)	14 (43.7%)	1.00
* Diarrhea in the attack before prophylaxis (n,%)*	9 (28.1%)	4 (12.5%)	0.120
* Constipation in the attack before prophylaxis (n,%)*	2 (6.2%)	1 (3.1%)	0.554
* Conjunctivitis in the attack before prophylaxis (n,%)*	1 (3.1%)	0 (0%)	0.313
Family history			
* Periodic fever syndrome (n,%)*	19 (59.3%)	21 (65.6%)	0.606
* Tonsillectomy (n,%)*	11 (34.3%)	15 (46.8%)	0.309
AIDAI score			
* First month before prophylaxis (Median (Min–Max))*	22 (0–72)	23 (0–80)	0.433
* First month after prophylaxis (Median (Min–Max))*	1 (0–144)	0 (0–45)	0.465
* Third month after prophylaxis (Median (Min–Max))*	0 (0–30)	0 (0–32)	0.674

*AIDAI* Auto-Inflammatory Diseases Activity Index, *MEFV* Mediterranean Fever

### Repeated measurements in pre-prophylaxis and post-prophylaxis period

**Fig. 1:**
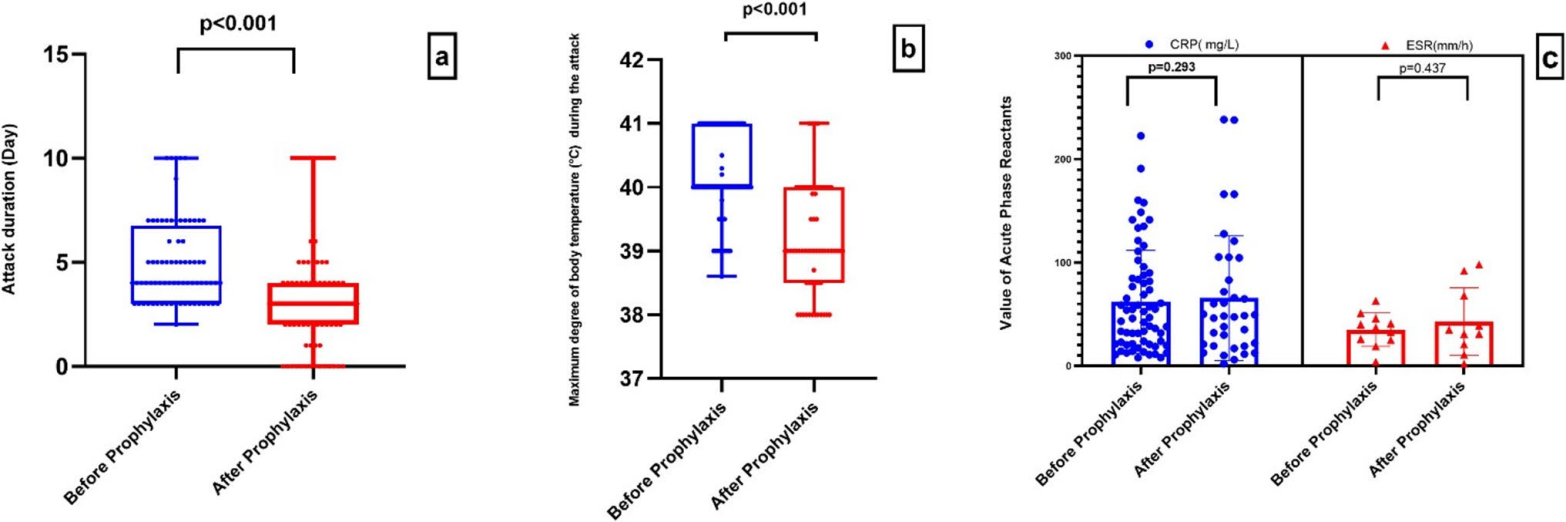
Repeated measurements in pre-prophylaxis and post-prophylaxis era. **a** Comparison of attack duration before and after colchicine treatment. **b** Comparison of maximum body temperatures during the attack before and after colchicine treatment. **c** Comparison of erythrocyte sedimentation rate (*ESR*) and C-reactive protein (*CRP*) values in the last attack before colchicine treatment and in the first attack after treatment

A statistically significant reduction was observed in both the median attack duration (4 (2–10)) vs. 3 (0–10) days; *p* < 0.001) and the median maximum body temperature (40.0 °C (38.6–41) vs. 39.0 °C (38–41); *p* < 0.001) when comparing the last attack before prophylaxis and the first attack after treatment initiation (Figs. 1a and 1b). In contrast, no significant differences were found in median CRP levels (46.9 (7.9–222.6) vs. 48.45 (2.1–238.3) mg/L; p = 0.293) or mean ESR values (35 ± 16.2 vs. 33 ± 2.82 mm/hour; p = 0.437) (Fig. 1c).

### Analysis of repeated measurement of AIDAI scores

**Fig. 2:**
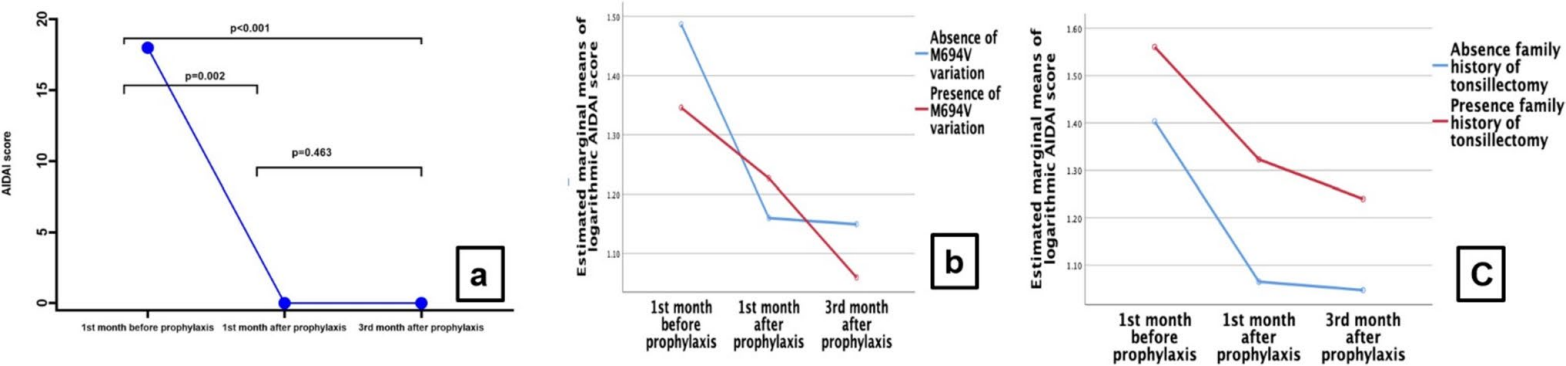
Change in AIDAI scores over time. **a** Decline in repeated AIDAI score measurements of patients before and after colchicine treatment. **b** Comparison of decreases AIDAI scores in patients with and without M694 V variation. **c** Comparison of decreases in AIDAI scores of patients with and without a family history of tonsillectomy

The mean monthly AIDAI score was 27.37 ± 19.45, 8.89 ± 19.70, and 5.66 ± 8.59 prior to colchicine initiation, at the first month, and at the third month following treatment initiation, respectively. Mean monthly AIDAI scores demonstrated a statistically significant reduction for the month preceding the initiation of colchicine treatment and for the first, and third months following colchicine treatment (*p* < 0.001). The difference between the mean AIDAI score in the month preceding the initiation of colchicine treatment and the mean AIDAI score in the 1 st month of treatment was statistically significant, whereas the difference between the mean AIDAI score in the 1 st month of treatment and the mean AIDAI score in the 3rd month of treatment was not statistically significant (*p* = 0.002 *vs. p* = 0.463) (Fig. 2a).

### Analysis of treatment response and its influencing factors

In evaluating treatment response, we concentrated on the alteration in AIDAI scores from the month preceding to the first month following the initiation of colchicine, termed Delta AIDAI, as this period exhibited the most pronounced reduction in disease activity. The analysis indicated that patients possessing the M694 V variant (F (2, 24) = 4.153, *p* = 0.028, partial η^2^ = 0.257), as well as those with a familial history of tonsillectomy (F (1.402, 22.427) = 5.308, *p* = 0.021, partial η^2^ = 0.249), demonstrated significantly smaller decreases in AIDAI scores over time (Table 2) (Figs. 2b and c).

**Table 2:** Evaluation of the factors affecting the change in AIDAI scores between the observation period of the study

		F	df1-df2	*p*	Partial Eta Square d (η2p)
Gender	Time	12.380	(1.429–22.861)	**0.001**	0.436
	Time/Gender	0.007	(1.429–22.861)	0.976	0.00
	Between groups	3.652	(1–16)	0.074	0.186
Age at the diagnosis	Time	4.870	(1.441–23.052)	**0.026**	0.233
	Time/Age at the diagnosis	0.657	(1.441–23.052)	0.480	0.039
	Between groups	0.295	(1–16)	0.594	0.018
Diagnostic delay	Time	3.174	(1.460–23.361)	0.074	0.166
	Time/Diagnostic delay	1.045	(1.460–23.361)	0.346	0.061
	Between groups	1.488	(1–16)	0.240	0.085
*Duration of attack	Time	1.275	(1.411–22.569)	0.287	0.074
	Time/Duration of attack	0.217	(1.411–22.569)	0.729	0.013
	Between groups	12.155	(1–16)	**0.003**	0.432
*Highest fever	Time	0.591	(1.403–22.454)	0.505	0.036
	Time/Highest fever	0.498	(1.403–22.454)	0.549	0.030
	Between groups	0.099	(1–20)	0.758	0.006
*GIS manifestation	Time	11.326	(1.446–21.264)	**0.001**	0.430
	Time/GIS manifestation	0.622	(1.446–21.264)	0.496	0.040
	Between groups	0.909	(1–15)	0.356	0.057
*Constitutional findings	Time	11.326	(1.446–21.264)	**0.001**	0.430
	Time/Constitutional finding	0.359	(1.446–21.264)	0.654	0.023
	Between groups	7.215	(1–15)	**0.017**	0.325
**CRP at diagnosis (mg/dl)	Time	6.746	(1.309–15.713)	**0.014**	0.360
	Time/CRP (mg/dl)	1.447	(1.309–15.713)	0.255	0.108
	Between groups	2.341	(1–12)	0.152	0.163
**ESR at diagnosis (mm/h)	Time	1.124	(2–6)	0.385	0.273
	Time/ESR(mm/h)	3.406	(2–6)	0.103	0.532
	Between groups	0.644	(1–3)	0.481	0.177
FH of periodic fever	Time	16.686	(1.422–22.756)	**0.00**	0.510
	Time/FH of periodic fever	1.524	(1.422–22.756)	0.237	0.087
	Between groups	0.234	(1–16)	0.635	0.014
FH of tonsillectomy	Time	3.352	(1.402–22.427)	0.068	0.173
	Time/FH of tonsillectomy	5.308	(1.402–22.427)	**0.021**	0.249
	Between groups	0.081	(1–16)	0.779	0.005
*MEFV* gen variation	Time	15.045	(2–24)	**0.00**	0.556
	Time/MEFV variation	1.391	(2–24)	0.268	0.104
	Between groups	0.116	(1–12)	0.739	0.010
M694 V variation	Time	9.072	(2–24)	**0.001**	0.431
	Time/M694 V variation	4.153	(2–24)	**0.028**	0.257
	Between groups	0.343	(1–12)	0.569	0.028
Exon 2 variation	Time	7.189	(2–24)	0.004	0.375
	Time/Exon 2 variation	0.399	(2–24)	0.676	0.032
	Between groups	0.987	(1–12)	0.340	0.076
Exon 10 variation	Time	14.155	(2–24)	**0.000**	0.541
	Time/Exon 10 variation	2.184	(2–24)	0.080	0.190
	Between groups	0.057	(1–12)	0.815	0.005

*CRP *C-Reactive Protein, *ESR *Erythrocyte Sedimentation Rate, *FH *Family History, *GIS *Gastro Intestinal System, *MEFV *Mediterranean Fever Variant

^*^During the attack before prophylaxis

^**^In the attack free period before prophylaxis

To further investigate factors influencing treatment response, linear regression analyses were conducted. The presence of the M694 V variant emerged as a significant negative predictor of AIDAI score improvement, both in univariate (B = −0.417, *p* = 0.007) and multivariate models (B = −0.399, *p* = 0.018) (Table 3).

**Table 3:** Analysis of the possible variables affecting the Delta AIDAI

Univariate Analysis	Unstandardized B	Coefficients Std. Error	Standardized Coefficients Beta	*t*	*p*	95% C.I for B
Gender	−0.005	0.137	−0.007	−0.036	0.972	−0.288–0.278
Age at the diagnosis	−0.001	0.003	−0.096	−0.480	0.636	−0.007–0.004
*GIS manifestation	−0.196	0.142	−0.266	−1.378	0.180	−0.490–0.097
M694 V variation	−0.417	0.139	−0.568	−3.011	**0.007**	−0.707 -(−0.127)
Exon 10 variation	−0.309	0.135	−0.465	−2.291	**0.034**	−0.591 -(−0.027)
Multivariate Analysis	Unstandardized B	Coefficients Std. Error	Standardized Coefficients Beta	*t*	*p*	95% C.I for B
Gender	−0.109	0.133	−0.165	−0.824	0.422	0.139–1.195
Age at the diagnosis	−0.001	0.003	−0.102	−0.459	0.652	−0.007–0.005
*GIS manifestation	−0.100	0.144	−0.151	−0.696	0.496	−0.591–0.101
M694 V variation	−0.399	0.151	−0.430	−2.648	**0.018**	−0.719-(−0.080)

*GIS* Gastrointestinal system

*During the attack

The distribution of patients according to their treatment response categories was as follows: 44 (57.9%) patients achieved a complete response, 15 (19.7%) patients exhibited a partial response, and 17 (22.4%) patients were classified as unresponsive to colchicine treatment.

### Analysis of factors affecting time to first attack after treatment

**Fig. 3:**
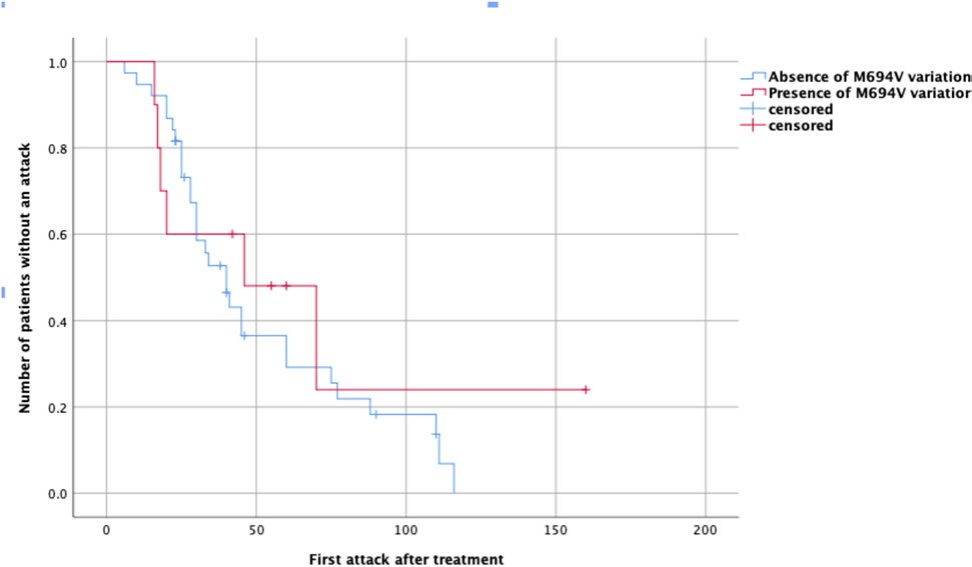
Assessing the effect of the M694 V variant on the interval between treatment initiation and the onset of the first attack

In Kaplan–Meier analyses conducted to evaluate the factors influencing the time to first attack after treatment, when stratified according to the presence of M694 V variation in the *MEFV* gene, no statistically significant difference was observed (Breslow test, *p* = 0.897), as illustrated in Fig. 3. Likewise, analyses stratified by gender did not demonstrate a significant difference in time to first attack (Breslow test, *p* = 0.853).

## Discussion

In this study, we analyzed the treatment response of patients with PFAPA receiving colchicine prophylaxis and the factors affecting treatment response using the AIDAI score. The most notable statistically significant reduction was observed in AIDAI scores between the one month before and one month after the colchicine initiation. Furthermore, we demonstrated that the M694 V variation has a negative effect on changing AIDAI scores by time.

The AIDAI is utilized to measure the activity of certain AIDs, and provides a standardized method to assess disease activity and monitor treatment response. Although it is not validated in PFAPA patients, an international consensus has stated that it can be employed in patients with PFAPA [14]. There is only one study that has evaluated the response to colchicine in PFAPA using AIDAI, and we regard this study as an accurate reflection of the patients’ respective disease statuses [13].

Since *MEFV* gene mutations are known to be associated with activation of the interleukin (IL)- 1β pathway, they have been investigated in inflammatory diseases in including Behçet’s disease, Crohn’s disease, ulcerative colitis and Immunoglobulin A vasculitis, and found to be associated with disease severity [16–19]. Several studies have shown increased serum or plasma levels of IL-1β, IL-6 and IL-18 during an attack in PFAPA. This finding suggests a role of inflammasomes in the pathogenesis of PFAPA, as in the diseases associated with *MEFV* gene variation [16–18]. Although the association between *MEFV* genotype and clinical presentation were widely investigated in PFAPA patients, data presented are contradictory [10, 20–22]. For instance, patients with *MEFV* gene variations were observed to experience shorter durations of attack; however, a subsequent study demonstrated that these attacks were more severe compared to those in individuals without such variations [23, 24]. In our study, initial disease severity, attack frequency, and attack duration were not significantly different between those with and without *MEFV* gene variation.

Although the relationship between *MEFV* variations and colchicine response were previously investigated, results are highly heterogeneous [10, 21, 25, 26]. In the present study, we showed that there was no significant difference between those with and without *MEFV* variation regarding the colchicine treatment response. However, the difference in AIDAI scores between the first month preceding treatment and the first month following treatment was found to be statistically significantly lower in patients with the M694 V variation compared to those without the M694 V variation. In addition, we demonstrated that the presence of *MEFV* gene variation does not influence the time length between treatment initiation and the first attack, as well. These conflicting results may also be associated with the varying treatment response definitions employed in different studies.

While our primary objective was to ascertain the"optimal"duration for treatment, our findings reveal that the most substantial clinical benefits are observed within the first month. The lack of significant improvements between the first and third months suggests that the majority of the treatment’s effects are realized early. Therefore, in clinical practice, if there is no complete response after the initial month, it may be necessary to reconsider the treatment strategy, which could involve adjusting the dosage or exploring alternative therapies. Nonetheless, further long-term research is necessary to more precisely determine the ideal treatment duration. The lack of studies examining the colchicine treatment duration on clinical improvement of PFAPA patients emphasizes the unique contribution of our study.

The primary limitation of this study is that the clinical symptoms during the diagnostic process were obtained from existing records rather than being collected contemporaneously during the study as a result of its retrospective design. Furthermore, an additional limitation is that our cohort comprised patients of Mediterranean descent, a population with a higher *MEFV* variation rate compared to others. Conversely, the principal strength of our study lies in our objective assessment of colchicine efficacy in PFAPA treatment utilizing the AIDAI, coupled with a comprehensive analysis of factors potentially influencing treatment response. Nevertheless, there is a lack of study assessing treatment efficacy in PFAPA utilizing the AIDAI score.

In conclusion, this study, which represents the first to evaluate the response to colchicine in PFAPA patients through repeated AIDAI scores, demonstrates that the majority of the treatment’s effects are evident within the initial month. Therefore, in clinical practice, if there is no substantial improvement after the first month, it may be necessary to reassess the treatment strategy, potentially by reviewing treatment adherence, modifying the dosage, or exploring alternative therapeutic options. However, given that the M694 V variation negatively impacts the change in AIDAI scores over time, it is suggested that the duration of treatment should be extended to adequately assess treatment response in patients with this variation.

## Supplementary Information

Below is the link to the electronic supplementary material.

Supplementary file1 (DOCX 19 KB)

## Data Availability

No datasets were generated or analysed during the current study.

## Abbreviations

AIDAI: Auto-Inflammatory Diseases Activity Index
*MEFV*: Mediterranean fever
PFAPA: Periodic fever, aphthous stomatitis, pharyngitis, adenitis syndrome
PRINTO: Paediatric Rheumatology International Trials Organisation

## Declarations

### The patient and public involvement statement

Patients or the public were not involved in the design, or conduct, or reporting, or dissemination plans of our research.
