# Supplementary material for: Colchicine treatment in PFAPA: how long should we wait for the clinical response?
Source: Eur J Pediatr. 2025 May 2;184(5):322. doi: 10.1007/s00431-025-06158-w (PMC12048419; doi:10.1007/s00431-025-06158-w)
Supplement: Supplementary file 1 — Supplementary file1 (DOCX 19 KB) [file 431_2025_6158_MOESM1_ESM.docx]

| Days | Fever  (>38 ^0^C) | Overall symptoms | Abdominal pain | Nausea/ vomiting | Diarrhea | Chest pain | Painful nodes | Arthralgia or Miyalgia | Swelling of the joints | Eyes manifestations | Skin rash | Pain relif drugs taken |
| --- | --- | --- | --- | --- | --- | --- | --- | --- | --- | --- | --- | --- |
|  |  | Oral aphthae |  |  |  |  |  |  |  |  |  |  |
|  |  | Sore throat/ Tonsillitis |  |  |  |  |  |  |  |  |  |  |
| Scored as: | 0/1 | 0/1 Yes/No | 0/1  Yes/No | 0/1 Yes/No | 0/1 Yes/No | 0/1 Yes/No | 0/1 Yes/No | 0/1 Yes/No | 0/1 Yes/No | 0/1  Yes/No | 0/1 Yes/No |  |
| 1) |  |  |  |  |  |  |  |  |  |  |  |  |
| 2) |  |  |  |  |  |  |  |  |  |  |  |  |
| 3) |  |  |  |  |  |  |  |  |  |  |  |  |
| 4) |  |  |  |  |  |  |  |  |  |  |  |  |
| 5) |  |  |  |  |  |  |  |  |  |  |  |  |
| 6) |  |  |  |  |  |  |  |  |  |  |  |  |
| 7) |  |  |  |  |  |  |  |  |  |  |  |  |
| 8) |  |  |  |  |  |  |  |  |  |  |  |  |
| 9) |  |  |  |  |  |  |  |  |  |  |  |  |
| 10) |  |  |  |  |  |  |  |  |  |  |  |  |
| 11) |  |  |  |  |  |  |  |  |  |  |  |  |
| 12) |  |  |  |  |  |  |  |  |  |  |  |  |
| 13) |  |  |  |  |  |  |  |  |  |  |  |  |
| 14) |  |  |  |  |  |  |  |  |  |  |  |  |
| 15) |  |  |  |  |  |  |  |  |  |  |  |  |
| 16) |  |  |  |  |  |  |  |  |  |  |  |  |
| 17) |  |  |  |  |  |  |  |  |  |  |  |  |
| 18) |  |  |  |  |  |  |  |  |  |  |  |  |
| 19) |  |  |  |  |  |  |  |  |  |  |  |  |
| 20) |  |  |  |  |  |  |  |  |  |  |  |  |
| 21) |  |  |  |  |  |  |  |  |  |  |  |  |
| 22) |  |  |  |  |  |  |  |  |  |  |  |  |
| 23) |  |  |  |  |  |  |  |  |  |  |  |  |
| 24) |  |  |  |  |  |  |  |  |  |  |  |  |
| 25) |  |  |  |  |  |  |  |  |  |  |  |  |
| 26) |  |  |  |  |  |  |  |  |  |  |  |  |
| 27) |  |  |  |  |  |  |  |  |  |  |  |  |
| 28) |  |  |  |  |  |  |  |  |  |  |  |  |
| 29) |  |  |  |  |  |  |  |  |  |  |  |  |
| 30) |  |  |  |  |  |  |  |  |  |  |  |  |
| 31) |  |  |  |  |  |  |  |  |  |  |  |  |

Name / Surname:

Age:

Month:

Year:

Each line represents a day in a month.

Please complete the diary **during all the time of the attacks** and score symptoms as yes (1) or no (0).

**Use a different diary for each month.** If you have no flair, bring back the diary empty.

Please note **only symptoms due to your auto-inflammatory disease**
